# Characterization and Prospective Applications of the Exopolysaccharides Produced by *Rhodosporidium babjevae*

**DOI:** 10.34172/apb.2020.030

**Published:** 2020-02-18

**Authors:** Rasool Mirzaei Seveiri, Masoud Hamidi, Cédric Delattre, Hamid Sedighian, Guillaume Pierre, Babak Rahmani, Sina Darzi, Clément Brasselet, Fatemeh Karimitabar, Ali Razaghpoor, Jafar Amani

**Affiliations:** ^1^Applied Microbiology Research Center, Systems Biology and Poisonings Institute, Baqiyatallah University of Medical Sciences, Tehran, Iran.; ^2^Department of Medical Biotechnology, Faculty of Paramedicine, Guilan University of Medical Sciences, Rasht, Iran.; ^3^Food and Drug Research Center, Vice-Chancellery of Food and Drug, Guilan University of Medical Sciences, Rasht, Iran.; ^4^Université Clermont Auvergne, CNRS, SIGMA Clermont, Institut Pascal, F-63000 Clermont-Ferrand, France.; ^5^Institut Universitaire de France (IUF), 1 rue Descartes, 75005 Paris, France; ^6^Department of Molecular Medicine, Faculty of Medicine, Qazvin University of Medical Sciences, Qazvin, Iran.; ^7^Department of Molecular Medicine, School of Advanced Technologies, Shahrekord University of Medical Sciences, Shahrekord, Iran.; ^8^Student Research Committee, Nursing and Midwifery Faculty, Qazvin University of Medical Sciences, Qazvin, Iran.

**Keywords:** Exopolysaccharides, *Rhodosporidium babjevae*, Antioxidant activity, Emulsifying activity, Antiproliferative activity

## Abstract

***Purpose:*** Due to the potential industrial and therapeutic applications of the yeast exopolysaccharides (EPSs), there has been an increasing demand to assess these biopolymers with improved characteristics. This study aimed to characterize the EPSs from *Rhodosporidium babjevae* (ATCC 90942 and IBRC-M 30088) as well as to evaluate their possible antioxidant, emulsifying and antiproliferative activities.

***Methods:*** *Rhodosporidium babjevae* was cultured for 5 days and following isolation of supernatant, EPSs precipitated with adding of cold absolute ethanol and freeze-dried. The EPSs chemical structure was determined by FT-IR, SEM, HPLC-SEC and GC-MS. Additionally the solubility, water holding capacity and emulsifying activity of EPSs were evaluated. In vitro, antioxidant activity was investigated against DPPH, superoxide and hydroxyl radicals. Finally the EPSs consequence on the cell proliferation of human breast adenocarcinoma (MCF-7) and Madin-Darby canine kidney (MDCK) cell lines was evaluated by 3-(4,5-dimethylthiazol-2-yl)-2,5-diphenyltetrazolium bromide (MTT) test.

***Results:*** *R. babjevae* excreted 1.6±0.2 g/L of the EPSs. The EPSs had three fractions with molecular weights of 1.02 ×10^6^ , 5×10^5^ and 2×10^5^ Da. Mannose and glucose were found as the main monosaccharides of the EPSs (84:16 mol%, respectively). The EPSs exhibited emulsifying activity on sun flower oil. The scavenging activities were found to be dose-dependent and higher than hyaluronic acid. Significant difference among the EPSs treatments on the proliferation of MCF-7 and MDCK cell lines was not observed (P>0.05).

***Conclusion:*** These results show the interesting potential of the EPSs from *R. babjevae* as biocompatible compounds for using in food and pharmaceutical fields.

## Introduction

Exopolysaccharides (EPSs) are high mass polymers secreted by form of microorganisms into the encompassing environment and are mainly made up of sugar residues.^1-3^ Sometimes, organic and inorganic functional groups such as amine, acetate, succinate, phosphate and sulfate can be seen in their chemical structures.^2^ Bacteria and fungi are among the most recognized EPSs producers.^1^ These compounds are considered as low-cost, non-toxic and natural biodegradable products which can be introduced as a suitable alternative to plant and algal products based on their characteristics.^1,2^

Besides bacteria, higher basidiomycetes and lower filamentous fungi, yeasts are also able to produce EPSs.^1^ Although, bacteria are the most common EPSs producers, the isolation procedure of EPSs produced by yeasts is easier and faster compared to bacterial EPSs; because of this reason, it can draw the attention to product on an industrial scale.^4^ So far, several studies have shown a wide range of biological activities of yeast EPSs including antioxidant, antitumor, antimutant, antithrombotic, anticoagulant, immunomodulation and antiviral activities.^1,5-8^ These activities are dependent on factors associated with the structure and physical characteristics of EPSs like their monomer compositions, molecular weights and presence of functional groups in their chemical structures.^1,2,5^ In addition to the mentioned descriptions, yeast EPSs have increasingly stimulated the interest in the food and pharmaceutical industries as an additive, bio-emulsifier, thickener and healing agent as well as may have a large potential markets in the mentioned industries.^1,5,6,9^ EPSs possess unique properties that are highly determined by the type of microorganism and the production conditions^1^; hence, each EPS requires a separate study to examine its biological activities.

Heretofore, several species of yeasts including *Aureobasidium*, *Bullera*, *Candida*, *Cryptococcus*, *Debaryomyces*, *Lipomyces*, *Pichia*, *Pseudozyma*, *Sporobolomyces* and *Rhodotorula* genera, are known for their ability to produce EPSs in laboratory culture conditions.^1,5^ Nevertheless, many of them have not been explored or are under investigation.

*Rhodosporidium babjevae* is a mucoid, red pigmented, non-ballistosporogenous and teliospore-forming yeast isolated from herbaceous plants and characterized by Golubev in Moscow region, Russia.^10^ Golubev has determined the preliminary monosaccharide composition of *R. babjevae*’s EPSs via paper chromatography, but there is a lack of studies on the physico-chemical properties and possible applications of the EPSs. However, the information on the properties of the extracted EPSs is still inadequate and no studies have been done regarding its characterization and biological activities. Thus, it is essential to explain both characterization and bioactivities of the EPSs.

As mentioned above, given the limited knowledge on the EPSs produced by *R. babjevae*, the monosaccharide composition, molecular weight, water holding capacity, antioxidant and emulsifying activity of the extracted EPSs were investigated to explore their potential in biotechnology. Additionally, the possible antiproliferative activity against human breast adenocarcinoma cell line (MCF-7 cells) was evaluated.

## Materials and Methods

### Microorganism and growth conditions

*Rhodosporidium babjevae* (IBRC-M 30088) was purchased from the Iranian biological resource center (IBRC) (equivalent to ATCC 90942). It was maintained by monthly transfers in potato dextrose agar and storing at 4°C. Pre-inocula were firstly prepared from cultures cultivated on potato dextrose agar. After 48 hours, each inoculating strain was cultured in 250 mL Erlenmeyer bottles (containing 50 mL of potato dextrose broth medium in each) on a rotary shaker for 48 hours at 180 rpm and 28°C. Aliquots of the related cultures were moved to 1000 mL Erlenmeyer bottles including 250 mL of basal medium for EPS production at a definitive concentration of 8% (v/v) and incubated for 120 h at 180 rpm and 28°C. The basal medium for EPS production contained 30 g/L glucose, 2.5 g/L (NH_4_)_2_SO_4,_ 1 g/L KH_2_PO_4,_ 0.5 g/L MgSO_4_.7H_2_O, 0.1 g/L NaCl, 0.1 g/L CaCl_2_.2H_2_O and 3 g/L yeast extract. The primary pH of the medium was adjusted to 5.5.^11^

### Recovery and purification of EPSs

The culture was centrifuged at 7900*×g* for 15 minutes at 4°C to separate cells from the supernatant. The EPSs in the collected supernatant were precipitated by adding two volumes of cold 96% ethanol and storing at 4°C for 24 hours. The precipitated EPSs were recovered through centrifugation at 7900*×g* for 12 minutes at 4°C and then were washed two times by adding cold 96% ethanol and centrifugation (7900*×g* for 12 minutes at 4°C). The EPSs were dissolved in deionized water and stored in a -80°C freezer. The frozen EPSs were dried using a Christ lyophilizer (Alpha 1-2 LDplus, Germany) till a fixed weight was seen and an exactness balance, which was used to determine the amount of EPSs, was achieved (grams of lyophilized EPSs/culture medium). Overall carbohydrate content of the EPSs was estimated by phenol-sulphuric acid technique using glucose as standard.^12^ The protein content of the EPSs was measured by the Bradford assay using bovine serum albumin as standard.^13^

### Characterization of EPSs solubility

The solubility of EPSs was tested in diverse solvents by previously reported method.^14^ According to this method, diminutive EPSs and different solvents (distilled water, methanol and chloroform) were mixed in 2 mL tubes, vortexed for 1 min and pellet dissolution was checked.

### Fourier transform infrared (FT-IR) spectroscopy

FT-IR spectroscopy is widely used for preliminary detection of the functional groups of EPSs. FT-IR spectrum of the EPSs was recorded on a Perkin Elmer spectrum two FT-IR system (USA). About 3 mg of the EPSs were dispersed on the universal attenuated total reflectance (UATR) and were analyzed using FT-IR spectrum measurement in wave number range of 400 to 4000 cm^-1^ (the IR spectra were recorded at room temperature). The obtained spectra were resulted of averaging 50 scans. The analysis of IR spectra was carried out by using spectrum ES software.

### Molecular weight determination

The molecular weight (Mw) of EPSs was estimated by HPLC (Agilent 1100 Series) steric exclusion chromatography (SEC) using TSKGEL PW-XL 3000 (10 μm, 7.8 x 300 mm) and TSKGEL PW-XL 5000 (10 μm, 7.8 x 300 mm) in series coupled to refractometer (RID). These two columns are preceded by a TSK gel PWXL pre-column (12 μm, 6.0 x 40 mm). Elution was performed at 30°C in isocratic mode by applying acetic acid 0.15 M + sodium acetate 0.1 M solution at a flow rate of 1 mL/min. A range of pullulan standards (Sigma-Aldrich) of different molecular weights was used from 1.3 kDa to 800 kDa. The EPSs and pullulan standards were injected (20 µL) at 10 g/L in elution buffer.

### Determination of EPSs monosaccharide composition

The monosaccharide composition of the EPSs produced by *R. babjevae* was analyzed using GC-MS. Briefly, the preliminary hydrolysis was carried out by dissolving 10 mg of the EPSs in 1 mL of 2 M trifluoroacetic acid. This mixture was vortexed for 10 sec and incubated in a dry water bath at 120°C for 90 min. The solution was evaporated under a stream of nitrogen. Trimethylsilylation derivatization was performed according to Pierre et al.^15^ l-Rha, l-Fuc, l-Ara, d-Xyl, d-Man, d-Gal, d-Glc, d-GlcA, d-GalA, d-GlcN and d-GalN, which were used, are standard. According to Benaoun et al,^16^ analysis was carried out by GC-MS-EI using Agilent 6890 Series GC System coupled to an Agilent 5973 Network Mass Selective Detector.

### Field emission scanning electron microscopy (FESEM)

In order to study the surface morphology and microstructure of the produced EPSs, micrographs were taken using a field emission scanning electron microscope (TESCAN MIRA3, Czech Republic) after gold coating with an accelerated voltage of 10–20 kV.

### Water holding capacity

To measure EPSs water holding capacity (WHC), experiment was performed as previously described by Niknezhad et al^17^ with a little modification. For this purpose, the EPSs (40 mg) were suspended and dispersed in 2 mL of distilled water and centrifuged at 11 000 rpm (40 min); afterward, the free water was discarded and the EPSs were dropped on a filter paper for full drain of water. Eventually, the EPSs (after water absorption) were weighed and noted. The percentage of WHC was calculated by the subsequent relation (Eq. 1):

WHC (%) = (Total EPSs weight after absorption)/ (Total dry EPSs weight) × 100 (Eq. 1)

### Emulsifying activity

Emulsifying activity was measured according to Priyanka et al^18^ with a little modification. The EPSs were dissolved in 5 mL distilled water (0.5%, 1% and 1.5% w/v) (at 40 to 42ºC) and placed in 15 mL polystyrene tubes, then 5 mL of sunflower oil (commercial brand) was added to every tube, vigorously mixed for 120 seconds to homogeneousness and left to stand for 24 hours at 4°C. Emulsifying activity was stated as the percentage of the height engaged by the emulsion layer respected to the entire height after 24 hours. Guar gum and sodium alginate were used as comparative emulsifiers at a concentration of 0.5% (w/v). The synergistic effect of the EPSs in relation to emulsifying activities was investigated in mixtures with guar gum and sodium alginate.

### Antioxidant activity

The free radicals (DPPH^•^, ^•^OH, O_2_^-•^) scavenging activities were calculated as the signs of antioxidant activity of the EPSs from *R. babjevae*. Different concentrations (0.1, 0.25, 0.5, 1, 2.5, 5 and 10 mg/mL) of the EPSs were used to assess the scavenging activities. The hyaluronic acid was utilized as a positive control for its power to scavenge the radicals.^19^

The DPPH^•^ scavenging activity was estimated as previously described by Niknezhad et al.^17^ According to this method, 50 µL of samples with different concentrations were mixed with 100 µL ethanol solution of DPPH (100 µM) in a 96 well plate. After placing in dark for 40 minutes at room temperature, the absorbance was noted at 525 nm and normalized to a blank sample (the blank contained only ethanol) and the control absorbance taken by the test of exchanging sample solutions with distilled water. The DPPH scavenging ability was calculated according to following equation (Eq. 2):

DPPH scavenging activity (%) = [(A_525nm_ of control - A_525nm_ of sample)/A_525nm_ of control] × 100 (Eq. 2)

The scavenging activity against ^•^OH radicals was determined by using FeSO_4_-salicylic acid method which was described by Niknezhad et al.^17^ Briefly, the reaction mixture was prepared by mixing 40 µL FeSO_4_ solutions (9 mM), 40 µL ethanol solution of salicylic acid (9 mM) and 40 µL samples with different concentrations. Afterward, 40 µL H_2_O_2_ solutions (8.8 mM) were blended with the mixtures to initiate the reaction. The final mixtures were incubated for 30 minutes at 37°C and the absorbance was noted at 510 nm. The calculation formula was similar to DPPH radical scavenging activity test. However, the absorbance of blank samples was revealed by replacing salicylic acid with distilled water.

The O_2_^-•^ scavenging activity was measured in the system of pyrogallol auto-oxidation in an alkalescent state. 40 µL of sample solutions with different concentrations were mixed with 120 µL of Tris-HCl buffer (50 mM, pH= 8.2). Then, 8 µL of 30 mM pyrogallol was inserted in the mixture, rapidly mixed and incubated at 25°C for 5 minutes. The reaction was finished by addition 20 µL concentrated HCl and the absorbance at 320 nm was recorded. The calculation formula was same with DPPH radical scavenging activity test. But, the absorbance of blank samples was determined by substituting pyrogallol with distilled water.

### Cell culture and antiproliferative activity of EPSs

Human breast adenocarcinoma (MCF-7 cell line) was used to assess the potential antiproliferative effect of the EPSs from *R. babjevae*. In this study, Madin-Darby Canine Kidney (MDCK) cell line was used as a model of normal cells. The cell lines were originally acquired from Pasteur Institute of Iran (Tehran, Iran). MCF-7 and MDCK cells were cultured in 25 cm^2^–culture flasks containing RPMI-1640 medium (Gibco, Karlsruhe, Germany) and Dulbecco’s Modified Eagle’s Medium (DMEM) (Gibco, Karlsruhe, Germany), respectively, which supplemented with 10% fetal bovine serum (Gibco, Karlsruhe, Germany), 100 U/mL penicillin and 100 µg/mL streptomycin (Sigma-Aldrich, Stockholm, Sweden), in a humidified atmosphere of 5% CO_2_ at 37°C. Aliquots of 1 mg of EPSs samples were dissolved and diluted with the media, generating doses of 20, 50, 100, 250, 500 and 1000 µg/mL. The antiproliferative actions of EPSs were determined by using 3-(4, 5-dimethylthiazol-2-yl)-2, 5-diphenyltetrazolium bromide (MTT) test. MTT assay is a colorimetric assay to assess cell viability and could reflect the dose-related toxicity.^20^

The cells (4 × 10^3^ cells/well) were seeded in 96-well plates with 100 µL of the media supplemented with 10% FBS and were allowed to attach overnight. Then, culture media were removed and cells were treated (triplicate) with EPSs solutions (20-1000 µg/mL) for 24 and 48 hours. Thereafter, the media were discarded and the MTT solution (0.5 mg/mL RPMI-1640 or DMEM) was added to each as well. After 4 hours of incubation, MTT solution was removed and the 100 µL of dimethyl sulphoxide (DMSO) solution was added to dissolve the formazan crystals. The optical density (OD) was measured on an ELISA plate reader (Bio Tech Instruments, USA) at 570 nm and data were normalized to a control group (cells that were only treated with the media). The percentage of viable cells was expressed as follows (Eq. 3):

Cell viability (%) = OD_T_/OD_C_ × 100 (Eq. 3)

Where: OD_T_ is the optical density measured for each treated group and OD_C_ is the mean of optical density detected for control group.

### Statistical analysis

All of the measurements stated in the present study were done in triplicate and the results were expressed as the mean ± SD. One-way analysis of variance (ANOVA) followed by a post hoc Tukey’s test was used to analyze the data (*P* < 0.05) using SPSS software version 16.0.

## Results and Discussion

### Purification and yield of EPSs

The EPSs yield from *R. babjevae* was 1.6 ± 0.2 g/L of the fermentation medium. The EPSs were composed of carbohydrate (78%) and protein (1.1%). Based on what was already mentioned in the present study, several genera of yeasts have been identified as EPSs producers. A study has been recently published on the ability of *Rhodosporidium paludigenum* to produce pullulan (a commercial polysaccharide).^21^ Except this one, no studies have been published on the extent of the production amount of EPSs in the *Rhodosporidium* genus and their applications, yet. The EPS production by *Candida boidinii* was reported to be lower than 1g/L.^22^ A locally isolated *Rhodotorula glutinis* strain yielded EPS with roughly yield of 0.8 g/L.^23^ In addition, the maximum amount of EPS produced by *Antrodia cinnamomea* (an edible Basidiomycete) was 0.49 g/L, after 14 days of fermentation.^24^ The production of EPSs in this study was higher than the production of EPSs in the above reports. In addition to the type of strain, factors such as culture conditions and the medium composition can affect the production of the EPSs^1^ and they need to be optimized. Therefore, in future studies, it is hoped that the production of the EPSs be scaled up for industrial applications.

### EPSs solubility

The freeze-dried EPSs was soluble in distilled water and insoluble in chloroform and methanol, and this was similar to the report available by Castellane et al.^14^ The aqueous solution of EPSs was clear and no sediment was observed after dissolution. The soluble nature of EPSs in water and non-dissolution in organic solvents is consistent with general properties of polysaccharides.

### FT-IR

**Figure 1:**
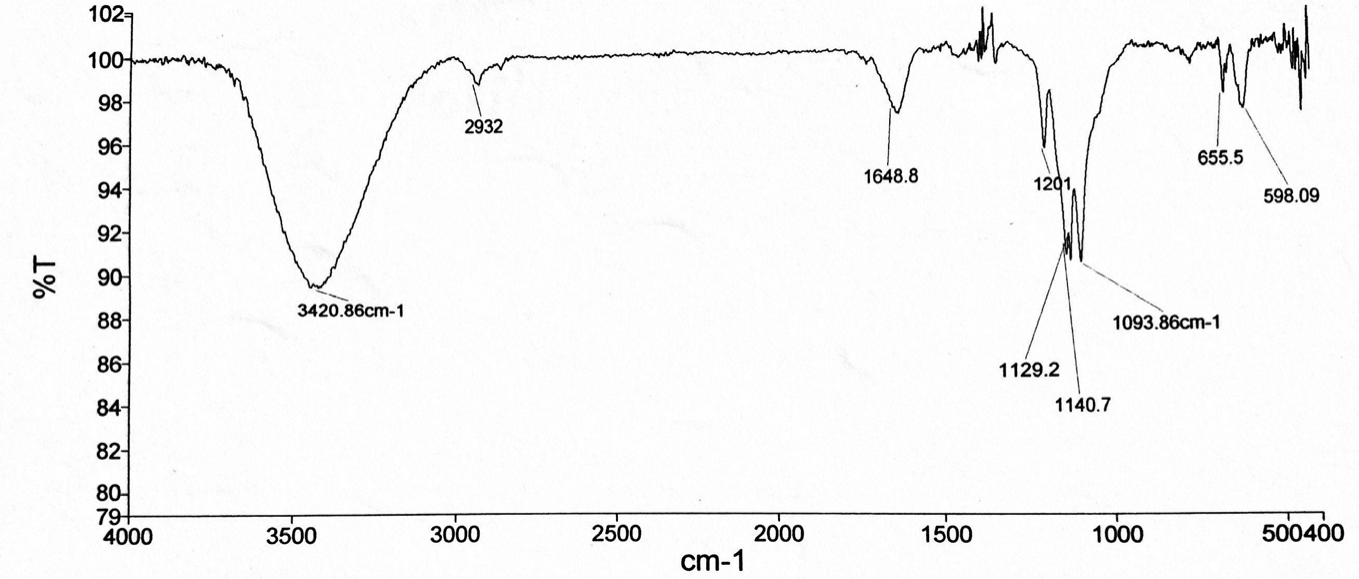
FT-IR spectrum of the EPSs produced by *Rhodosporidium babjevae*.

The FT-IR spectrum of the EPSs is presented in Figure 1, which display a variety of typical absorption bands of polysaccharides. The broad and intense absorption band at 3420.86 cm^-1^ was due to stretching of –OH (hydroxyl) groups. The weak band at 2932 cm^-1^ was corresponded to the aliphatic C-H (sp^3^) stretching. The band in the region of 1648.8 cm^-1^ was characteristic of the C═O stretching. A peak at 1201 cm^-1^ suggested that the EPSs may have a pyranose ring.^25^ The absorption bands at 1093.86 cm^-1^, 1129.2 cm^-1^ and 1140.7 cm^-1^ were corresponded to the C-O stretching vibrations and might be attributed to the ether group of polysaccharides or could be assigned to beta-glycosidic linkages.^4^ The absence of peak at 1726 cm^-1^ demonstrated that the EPSs had no uronic acid.^25^

### Molecular weight and monosaccharide composition analyses

The molecular weight distribution of the EPSs was determined by HPLC-SEC using RI detection. The monosaccharide composition of the EPSs was analyzed through GC-MS. The results showed that the EPSs were composed of mannose (84%) and glucose (16%), and three fractions with different molecular weights were observed as well; the elution profile exhibited main fraction with a molecular weight estimated as 1020 kDa, based on pullulan standards (Table 1).

**Table 1:** Monosaccharides composition and molecular weight distribution of the EPSs extracted from *Rhodosporidium babjevae*

**Monosaccharides (mol%)** ^*^			**Mw (g/mol)** ^**^
Glc	Man	Glc/Man	1.02 ×10^6***^, 5×10^5^ and 2×10^5^
16	84	0.2	

* Monosaccharides composition was estimated by GC/MS analysis. Glc: glucose, Man: mannose. ** Mw: molecular weight estimated by HPSEC with RI detection using pullulan standardization. All analyses were run in triplicate and the relative standard deviations are less than 5%. *** Main fraction.

Previously, Golubev had a slight mention on the EPSs produced by *R. babjevae,*^10^ but there were serious weaknesses in his study. He only identified the monosaccharide composition of the EPSs using paper chromatography which is a trivial method for this purpose, while GC-MS is a more accurate and reliable technique than paper chromatography. He did not also mention the molecular weight or yield of the EPSs in his study. According to Golubev, the EPSs were composed of mannose, galactose, glucose and fucose^10^; which is in conflict with our findings. This difference may be due to the different medium components (including sources of carbon and nitrogen) which can affect the EPSs produced by the yeasts.^5^ Golubev cultivated the yeast in a medium that contained only glucose, yeast extract and peptone water. The glucose (as carbon source) and yeast extract (as nitrogen source) were same with our study, but the fermentation medium used in our study also contained another source of nitrogen (ammonium sulfate) which is commonly used in yeast culture media to produce EPSs.^1,5^

So far, EPSs are produced by a considerable number of yeasts.^1^ According to Grigorova et al the monosaccharide composition of EPS produced by the *Rhodotorula acheniorum* MC was only mannose and glucose, with the ratios of 92.8% and 7.2%, respectively.^26^ According to Cho et al, the EPS produced by *Rhodotorula glutinis* KCTC 7989, is consisted of four monosaccharides, mannose, fucose, glucose and galactose, accounting for 6.7:0.2:0.1:0.1 and this EPS had an average molecular weight of 100-380 kDa.^27^ Study by Pavlova et al showed that the EPS produced by *Cryptococcus flavus* AL51 was a neutral polysaccharide composed of mannose, glucose, xylose and galactose with the ratios of 55.1:26.1:9.6:1.9 which had a molecular weight equal to 1010 kDa.^28^ Breierová et al showed that the EPSs produced by *Cryptococcus laurentii* CCY 17-3-16 were rich in mannose and had three fractions with different molecular weight distribution.^29^ Our results showed that the EPSs produced by *R. babjevae* were rich in mannose, similar to the studies mentioned in this section and had three fractions with different molecular weight distribution.

Also it is worth noting that among the known industrial polysaccharides, glucomannan and galactomannan are structurally similar to the EPSs obtained from *R. babjevae*. These two important polysaccharides are mainly derived from plant sources.^30,31^ Konjac glucomannan mainly contains mannose and glucose (similar to the EPSs from *R. babjevae*) and has many health benefits (such as anti-obesity, anti-diabetic, anti-inflammatory, laxative and prebiotic activities).^31^ Due to konjac glucomannan’s good water absorptivity, thickening, emulsifying, stability and film-forming properties, it is used as an additive in various products (pharmaceutical, food and biotechnological products).^30^ Despite of these properties, prolonged consumption of konjac glucomannan can lead to complications such as esophagus blockage and gastrointestinal distress.^31^ In addition, as Mahapatra and Banerjee stated in their paper, the microbial EPSs that are almost similar to industrial polysaccharides (obtained from plants) can be alternatives to the plant polysaccharides (because of large production in short time period, easy isolation and purification).^1^ Overall, further studies on the EPSs from *R. babjevae* are recommended.

### Morphology and water holding capacity of EPSs

**Figure 2:**
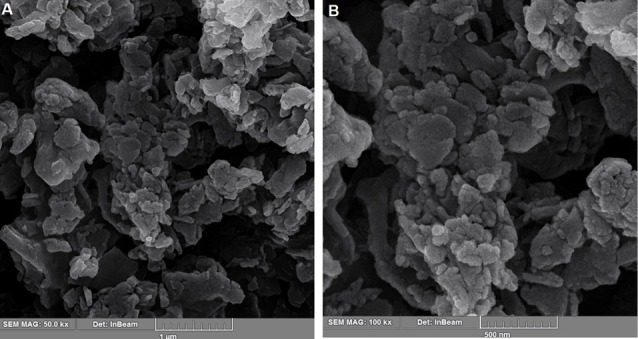
FE-SEM showing the surface morphology of the EPSs produced by *Rhodosporidium babjevae* at × 50000 **(A)** and ×100000 **(B)** magnification.

Field emission scanning electron microscopy was a known technique for studying the microstructure of EPSs.^32^ As shown in Figure 2A-B, the EPSs micrographs are presented a porous and three-dimensional structure and several crevices were observed in their microstructure. There were many studies on microstructures of EPSs which some of them have reported the porous nature of EPS.^32-34^ Ramirez suggested that EPS of porous nature can be used as a thickener, texturizer, viscosifier and stabilizing agent in food industry.^4^

The water holding capacity of the EPSs was 519 ± 18.5%. This was greater than that of EPS produced by *Leuconos toclactis* KC117496 and *Lactobacillus keﬁranofaciens* ZW3 (117% and 496%, respectively).^35,36^ Majumder and Goyal found that the pores in EPS microstructure enable it to hold water through hydrogen bonds.^34^ Given this phenomenon, it can be guessed why the EPSs have a high ability to hold water. Zhu et al showed that an EPS from *Bacillus atrophaeus* WYZ strain has a porous microstructure and can be used in the field of drug delivery and biomaterials.^37^ The existence of porous microstructure in the EPSs might be desirable for their application in the drug delivery systems as well as an excipient in the pharmaceutical industry.

### Antioxidant activity

**Figure 3:**
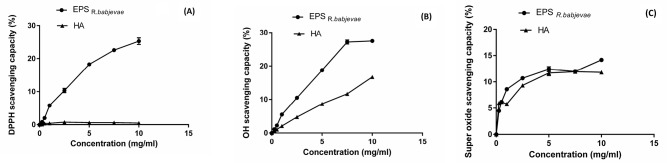
The DPPH radical (A), hydroxyl radical (B) and superoxide anion radical (C) scavenging activities (capacities) of the EPSs from *Rhodosporidium babjevae* with hyaluronic acid as the positive control.

The DPPH^•^, ^•^OH, O_2_^-•^ scavenging rates of EPSs from *R. babjevae* were gradually increased by increasing the concentrations. The results showed that the scavenging rates for DPPH^•^, ^•^OH and O2^-•^ of the EPSs reached to 25.2 ± 1%, 27.5 ± 0.5% and 14.1 ± 0.2% at the concentration of 10 mg/mL, respectively, indicating the EPSs showed higher antioxidant activity than those of hyaluronic acid (Figure 3A-C), an important biopolymer in cosmetics that can scavenge free radicals. Osińska-Jaroszuk et al extracted a crude EPS from the white rot fungus *Ganoderma applanatum* and showed its DPPH^•^ scavenging activity that did not exceed more than 20%.^38^ Ma et al extracted EPSs from *Rhodotorula mucilaginosa* CICC 33013, which is a yeast strain obtained from the China Center of Industrial Culture Collection (CICC).^39^ The authors proved that the DPPH^•^ scavenging activities of the crude EPS reached to about 60% at the concentration of 8 mg/mL. Sun et al studied the antioxidant activities of the EPS from the arctic marine bacterium *Polaribacter* sp. SM1127; it has been reported that the DPPH^•^, ^•^OH, O_2_^-•^ scavenging activities of the EPS at a concentration of 10 mg/mL were 55.4 ± 3%, 52.1 ± 2.1% and 28.2 ± 3%, respectively, which is similarly higher than those of hyaluronic acid.^40^

Free radicals can greatly affect human health and cause various diseases such as ageing and inflammation in the body^41^; for this reason, researchers are always looking for biopolymers that are antioxidant and can reduce the harm caused by free radicals in the human body. Sun et al suggested that the ability of an EPS in scavenging of free radicals may be due to the presence of functional groups such as -O-, C=O and –OH in its structure.^40^ It is well accepted that the mentioned functional groups can donate electron or hydrogen ions to reduce the free radicals and can bind directly with the free radicals to form stable radicals.^40,42^ As shown previously in the spectrum obtained from FT-IR, the existence of the mentioned functional groups in the EPSs structure was confirmed. Zhu et al suggested that the mannose content of EPS may play a role in its antioxidant activity.^43^ In addition, according to Chen et al the antioxidant activities of EPSs were not dependent on a single factor and were the result of coincidence of many factors (such as the ratios of monosaccharide, molecular weight and glycosidic branching); Also, the antioxidant mechanism of EPSs is not clearly understood.^44^ However, the precise antioxidant mechanism of the EPSs has not yet been fully disclosed.

### Emulsifying activity

The EPSs could emulsify sun flower oil 0.5%, 1% and 1.5% at 4°C (Table 2). The emulsions produced by the EPSs were soft and semi-solid gel emulsions. The results showed that the emulsifying activity of the EPSs increased from 48.7 ± 2.3% to 54.7 ± 1.6% when the amount of EPSs increased and they were more efficient than that of sodium alginate, at the same concentration (0.5% w/v). A large number of microbial EPSs were capable of producing oil in water emulsions.^9,45^ According to Sahana et al the ability of an EPS to emulsify oils depends on several factors such as its carbohydrate moiety and attached protein moiety.^46^ In the process of EPSs’ emulsification, carbohydrate moiety plays an important role in the aqueous phase, the hydrophobic residues of protein moiety react with the oil phase and finally, emulsion is created.

**Table 2:** Emulsifying activity of the EPSs synthesized by *Rhodosporidium babjevae* and commercial hydrocolloids

**EPSs and hydrocolloids Concentration (% in water medium)**	**Emulsifying activity (%)**
0.5 % EPSs	48.7 ± 2.3
1% EPSs	53.7 ± 0.6
1.5% EPSs	54.7 ± 1.6
0.5% Sodium alginate	42.3 ± 1.2
0.5% Guar gum	72.3 ± 1.5
1% EPSs + 0.5% Sodium alginate	62 ± 2
1% EPSs + 0.5% Guar gum	88 ± 2

Also, the synergistic effect of sodium alginate and guar gum on the emulsion produced by the EPSs was observed. Accordingly, the emulsifying activity of the EPSs alone (1% w/v) was increased from 53.7 ± 0.6% to 62 ± 2% and 88 ± 2% followed by adding the sodium alginate and guar gum (0.5% w/v), respectively. Similarly, several studies have shown the synergistic effect of commercial hydrocolloids on the emulsions produced by microbial EPSs.^9,45^

Altogether, these results show that the EPSs produced by *R. babjevae* have good emulsifying activity for oil-water emulsion systems, independently or in combination with other commercial polysaccharides.

### Antiproliferative activity

**Figure 4:**
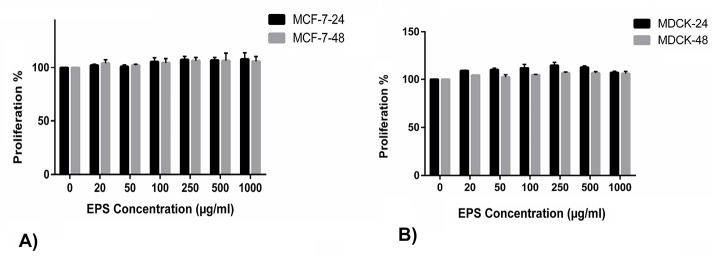
Proliferation (survival) activity of the EPSs from *Rhodosporidium babjevae* against human breast adenocarcinoma (MCF-7) cell line **(A)** Madin-Darby Canine Kidney (MDCK) cell line **(B)** after 24 hours and 48 hours incubation. Each value is expressed as mean ± SD (n=3).

There were no significant variations within the cell proliferation of MCF-7 cell line among six concentrations (20 to 1000 µg/mL) of the EPSs treatments after 24 and 48 hours, separately. Also, the cell proliferation within the MCF-7 cell line did not considerably differ between 24 and 48 hours under any of the matched EPSs treatments (Figure 4). The cell viability of MDCK cell line was similar in control condition as well as each of the six concentrations of EPSs after 24 and 48 hours, separately. Compared to the control condition, the cell viability in the MDCK cell line was not considerably totally different between 24 and 48 hours under every of the matched EPSs treatments (Figure 4).

Evidences regarding the antiproliferative properties of the EPSs from *Rhodosporidium* genus are lacking. In this research, the EPSs from *R. babjevae* did not show significant changes versus proliferation of MCF-7 cell line. Ruiz-Ruiz et al showed that EPS yielded by *Halomonas stenophila* had not any significant antiproliferative effect against MCF-7 as well as human T leukemia cell lines, but the over sulfated form of this EPS (modified with sulfate saturation method) exhibited a higher growth inhibition on the tumor cells.^47^ It has been previously documented that creating chemical modifications as over sulfation on natural polymers could improve their biological activities via changing the negative charges of the EPS for interacting with the cell targets.^47,48^ The EPSs studied in the present study were native biopolymers and no chemical modifications have been made on their structures. Our findings are in contrast with the results of Ma et al in which high molecular weight REPS2-A (an EPS with molecular weight equal to 7.125×10^6^ Da) produced by *Rhodotorula mucilaginosa* CICC 33013 exhibited high growth inhibition on HepG2 cell line.^39^ Ruiz-Ruiz et al suggested that viscosity of the EPS could play an important role in preventing the proliferation of cancerous cells^47^ and it is well-known that viscosity has a direct relationship with molecular weight. The molecular weights of our EPSs were lower than that of REPS2-A.

The present findings showed that the EPSs exhibited no antiproliferative activity against the MDCK cell line. In consistent with our findings, several studies have shown the non-toxic nature of EPSs on different cell line models.^46,49,50^ Regarding the no cytotoxicity effect of the EPSs on normal cell line of MDCK, it seems they have biocompatibility features. The biocompatibility is one of the desirable properties of the natural polymers for use in the formulation of food products, medicines and cosmetics. Biomaterials with these features can be good candidates for use in the development of tissue engineering scaffolds and there are recent studies conducted on the use of EPSs in the preparation of the scaffolds.^51,52^ Therefore, more detailed research on the use of the EPSs in the development of new scaffolds is recommended.

## Conclusion

This work was reported on *R. babjevae* capable of producing 1.6±0.2 g/L of EPSs. The monosaccharide composition of the EPSs was mannose and glucose (84:16) with a ratio of 5.25. The EPSs exhibited three fractions which the main fraction had a high molecular weight equals to 1020 kDa. The microstructure was three-dimensional and porous, and the EPSs showed significant water holding capacity. The EPSs showed a relatively good emulsifying ability as well as exhibited free radical scavenging activity (higher than that of hyaluronic acid). In addition, the EPSs had not any antiproliferative effect and can be applied as biocompatible compounds. These indicate that the EPSs have the potential as safe biomaterials. However, further studies exploring their chemical structures and physical properties such as glycosyl linkages and rheological properties as well as their other applications in more details are recommended.

## Ethical Issues

Not applicable.

## Conflict of Interest

The authors of this study have declared no conflict of interest.

## Acknowledgments

This work was supported by the applied microbiology research center, Baqiyatallah University of Medical Sciences, Tehran, Iran. The authors thank Shaghayegh Pishkhan Dibazar and Dariush Ghasemi for technical assistance.
